# Cerebellopontine Angle Lipoma Associated to Dysplastic Labyrinth

**DOI:** 10.5334/jbsr.1472

**Published:** 2018-04-20

**Authors:** Martin Gombert, Patrick Mailleux

**Affiliations:** 1 Saint Luc Bouge, BE

**Keywords:** lipoma, Cerebellopontine angle lipoma, intracranial lipoma, vestibulocochlear lipoma, MRI, CT

A 14-year-old female patient was referred to our radiology department for evaluation of right hypoacousia. Unenhanced Computed Tomography (CT) of the temporal bones showed a 15 mm hypodense mass infiltrating the right cerebellopontine angle (CPA) and the internal auditory canal (IAC) (arrows, Figure 1A). This mass showed negative Hounsfield Unit (HU) values in keeping with fatty content. Vestibulo-cochlear malformations were also found, including a hypoplastic lateral semi-circular canal (arrows, Figure 1B) and a globular aspect of the cochlea with an underdeveloped third turn (not shown).

**Figure 1:**
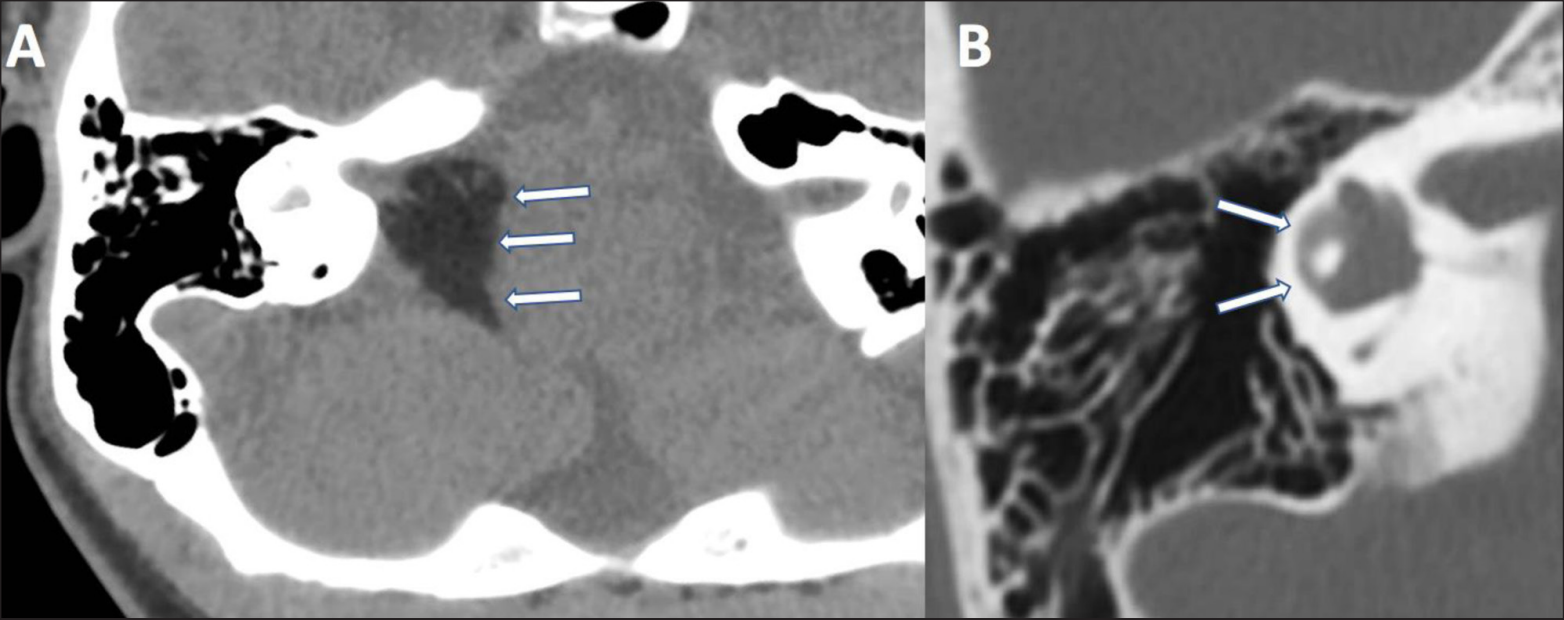


Magnetic Resonance Imaging (MRI) performed for better evaluation showed homogeneous high T1 signal of the lesion with signal drop on fat-saturated sequence, thus confirming the fatty composition of the mass (curved arrows, Figure 2B).

**Figure 2:**
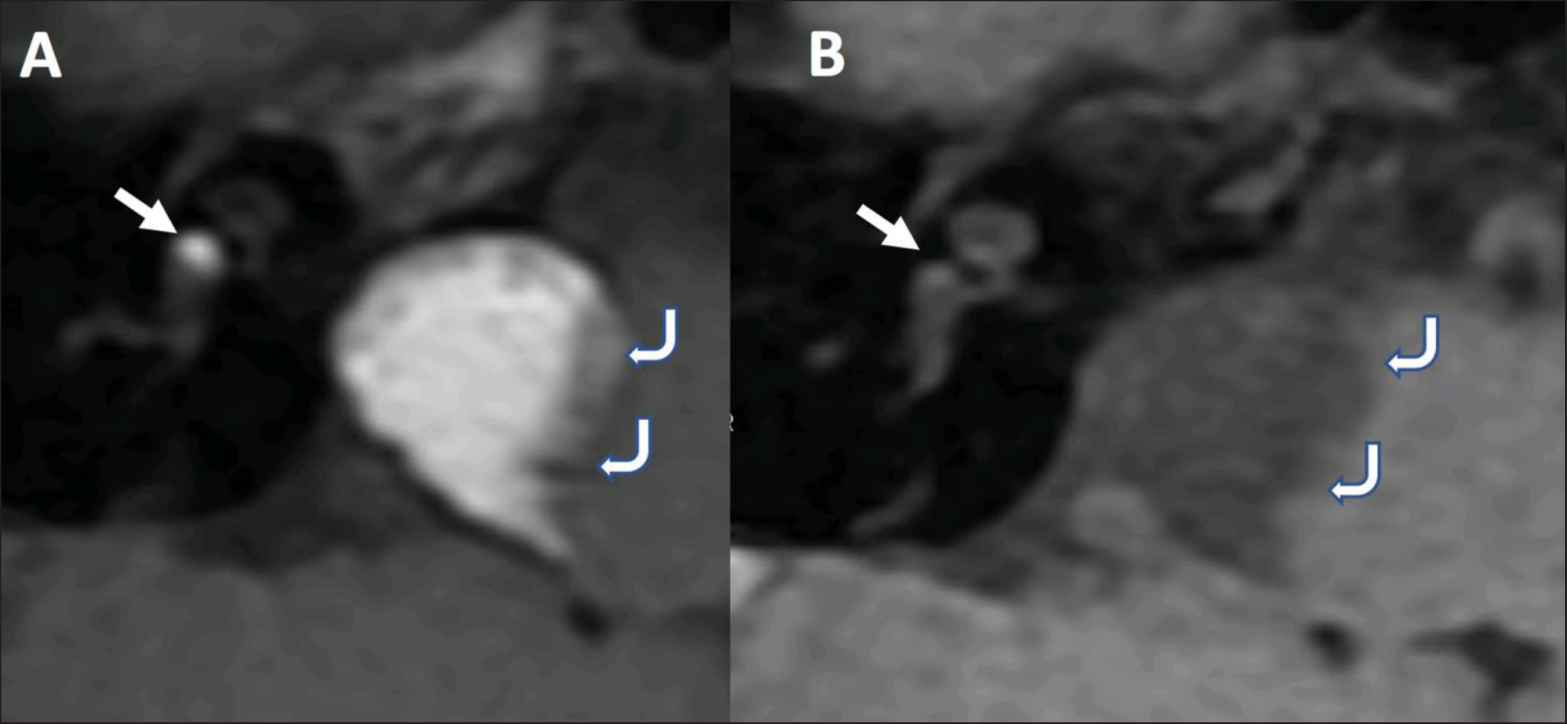


The T1-weighted images also highlighted two more fatty deposits in the right labyrinth with the first one located in the basal turn of the cochlea and the second in the vestibule (arrows, Figure 2). There was no evidence of mass effect on the brainstem on T2-weighted images, and the facial and vestibulo-cochlear nerves could be easily identified crossing through the mass with only slight angulation (curved arrows, Figure 3).

**Figure 3:**
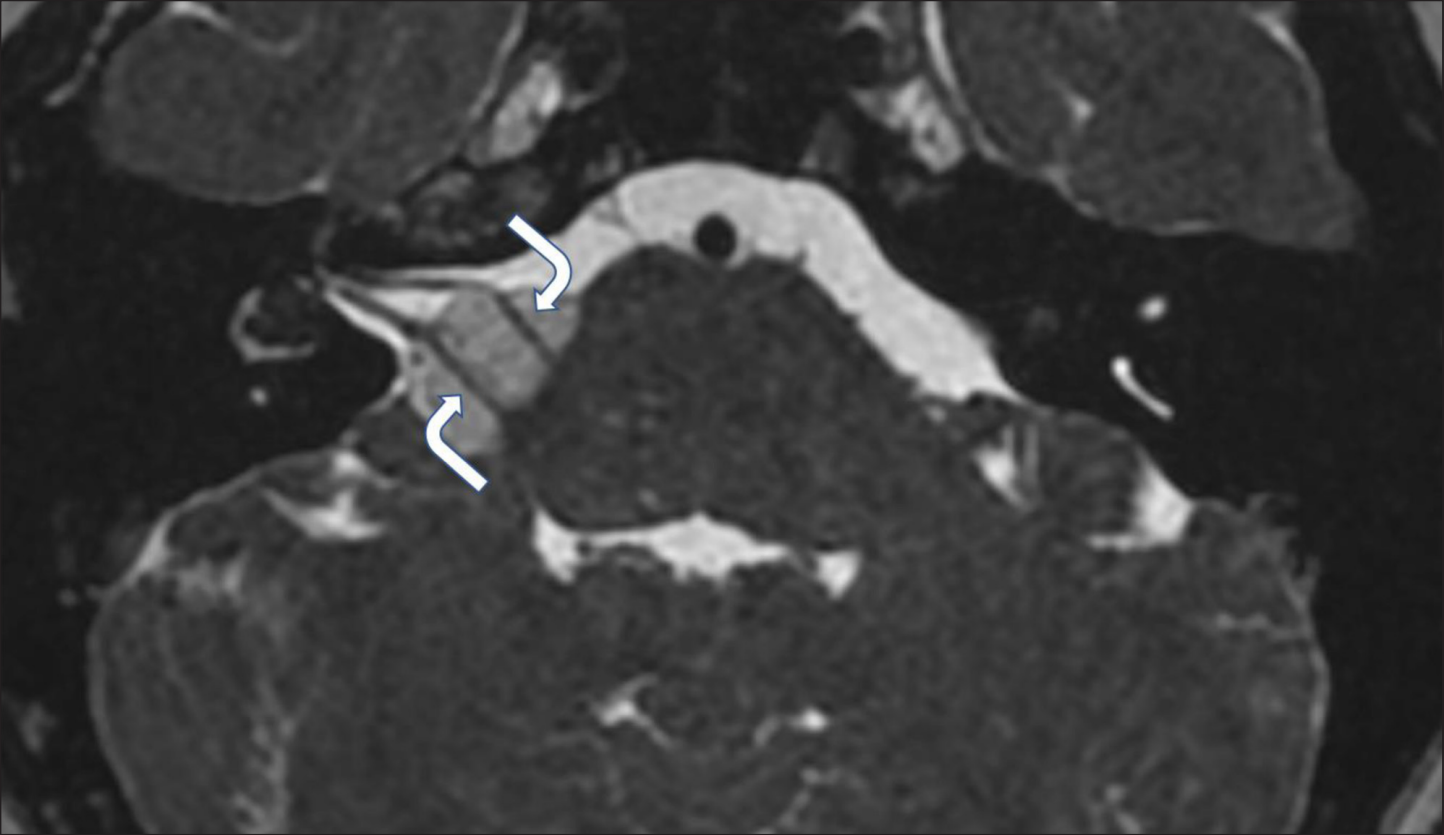


Based on these findings, the diagnosis of cerebello-pontine angle lipoma was made. The patient was referred to otorhinolaryngologist for conservative treatment.

## Comment

Cerebellopontine angle lipoma is a very rare lesion representing 0.1% of all the CPA tumors [1]. CPA Lipomas are usually asymptomatic, although some patients can present with progressive slow hearing loss, dizziness, trigeminal symptoms or facial para-paresis.

CT typically shows a well-defined homogenous low attenuation mass with negative HU values. On MRI, the fatty composition of the lipoma is easily recognized by demonstrating a homogenous high-intensity T1 signal with signal drop on fat-saturated sequences. No enhancement is observed after intravenous contrast medium injection. Furthermore, unlike other CPA tumors, facial and vestibulo-cochlear nerves can be observed crossing through the lipoma, with no significant mass.

Differential diagnosis includes a ruptured intracranial dermoid cyst as it also contains a significant amount of fat. However, in that case, the primary lesion would usually be identified in the midline with multiple droplets within the subarachnoid space.

Finally, careful evaluation should be conducted to identify any associated malformations (i.e. vestibulocochlear malformations and lipomas) since CPA lipoma is considered a developmental disorder. Surgery should only be considered for symptomatic patients in whom conservative treatment has failed given that lipoma is a non-neoplasic slowly growing lesion closely related to nervous structures [1].
